# Long-term effects of air pollutants on respiratory and cardiovascular mortality in a port city along the Adriatic sea

**DOI:** 10.1186/s12890-023-02629-8

**Published:** 2023-10-18

**Authors:** Federico Mei, Matteo Renzi, Martina Bonifazi, Floriano Bonifazi, Nicola Pepe, Alessio D’Allura, Giuseppe Brusasca, Giovanni Viegi, Francesco Forastiere

**Affiliations:** 1 Department of Biomedical Sciences and Public Health, Marche Polytechnic University, Ancona, Italy; 2 Respiratory Diseases Unit, Azienda Ospedaliero-Universitaria “Ospedali Riuniti”, Ancona, Italy; 3 Department of Epidemiology of Lazio Region, ASL Roma 1, Rome, Italy; 4 Honorary President Associazione Allergologi Immunologi Italiani Territoriali E Ospedalieri, , Firenze, Italy; 5 ARIANET S.R.L., Milan, Italy; 6 Institute of Clinical Physiology, National Research Council (CNR), Pisa, Italy; 7 Institute of Translational Pharmacology, National Research Council (CNR), Palermo, Italy; 8 Environmental Research Group, Imperial College, London, UK

**Keywords:** Mortality, WHO AQGs, Nitrogen dioxide, PM_2.5_, PM_10_, Port

## Abstract

**Background:**

Shipping and port-related air pollution has a significant health impact on a global scale. The present study aimed to assess the mortality burden attributable to long-term exposure to ambient particulate matter (PM_2.5_, PM_10_) and nitrogen dioxide (NO_2_) in the city of Ancona (Italy), with one of the leading national commercial harbours.

**Methods:**

Exposure to air pollutants was derived by dispersion models. The relationship between the long-term exposure of air pollution exposure and cause-specific mortality was evaluated by Poisson regression models, after adjustment for gender, age and socioeconomic status. Results are expressed as percent change of risk (and relative 95% confidence intervals) per 5 unit increases in the exposures. The health impact on the annual number of premature cause-specific deaths was also assessed.

**Results:**

PM_2.5_ and NO_2_ annual concentrations were higher in the area close to the harbour than in the rest of the city. Positive associations between each pollutant and most of the mortality outcomes were observed, with estimates of up to 7.6% (95%CI 0.1, 15.6%) for 10 µg/m^3^ increase in NO_2_ and cardiovascular mortality and 15.3% (95%CI-1.1, 37.2%) for 10 µg/m3 increase PM_2.5_ and lung cancer. In the subpopulation living close to the harbour, there were excess risks of up to 13.5%, 24.1% and 37.9% for natural, cardiovascular and respiratory mortality. The number of annual premature deaths due to the excess of PM_2.5_ and NO_2_ exposure (having as a reference the 2021 World Health Organization Air Quality Guidelines) was 82 and 25, respectively.

**Conclusions:**

Our study confirms the long-term health effects of PM and NO_2_ on mortality and reveals a higher mortality burden in areas close to shipping and port-related emissions. Estimating the source-specific health burdens is key to achieve a deeper understanding of the role of different emission sources, as well as to support effective and targeted mitigation strategies.

## Introduction

Human health effects of exposure to ambient air pollution, including particulate matter (PM) and gaseous contaminants, are a primary public concern, as they are responsible for approximately 3 million premature deaths per year globally [1].

Particulate matter (PM) is made of a complex mixture of chemicals with different compositions, physical features, sources and emission profiles [2]. The health impact of mass concentrations of PM with aerodynamic diameter ≤ 2.5 μm (PM_2.5_, mainly originated from combustion sources) and with ≤ 10 μm (PM_10_, composed also of crystal material, sea salt and biological material) has been largely investigated over the last decades [3]. The overall evidence suggests a causal relationship between PM_2.5_ and morbidity and mortality, especially from cardiovascular and respiratory conditions [4]. An additional air pollutant with established toxicity for human health is nitrogen dioxide (NO_2_), considered a key precursor of secondary pollutants, as newly generated organic, nitrate, and sulfate particles contribute to the PM_10_/PM_2.5_ mass.

Due to the increasing body of evidence suggesting that air pollution affects human health at lower concentrations than previously thought, the World Health Organization (WHO) has recently updated the air quality guidelines (AQGs). In detail, PM_2.5_, PM_10_, and NO_2_ annual guidelines have been lowered respectively to 5, 15 and 10 µg/m^3^ [2].

Air pollutant concentrations are influenced by interaction among different factors, including weather conditions, diurnal/seasonal cycles in solar radiation, local sources and emission rates [5]. Urban areas are significant contributors and the more appropriate unit of analysis, especially where intense industrial and portal activities are present [6, 7]. With reference to shipping and port-related emissions due to maritime transport, the International Maritime Organisation (IMO), by means of the International Convention for the Prevention of Pollution from Ships (MARPOL) Annex VI, provided the regulations for reducing air pollution and established emission control areas (ECA) for sulphur oxides (SOx) and nitrogen oxides (NOx) [8], which were revised over time, further lowering fuel content [9]. However, despite the IMO emission control measures, there are still vast amounts of emissions from shipping, ships at berth and port activities, and the relative health burden was estimated to have further increased, with ~ 265,000 global premature deaths projected for 2020 (i.e. 0.5% of global mortality), due to rapid increases in maritime transport volumes [10]. This literature review, focused on the health impact attribultable to shipping and port-related emissions, included 32 articles on this topic, but, among these, no investigations have specifically assessed differences in health outcomes according to distance from the port [10].

The Italian city of Ancona is located in the center-east part of the country along the sea and has a leading national commercial harbour, placed very close to the center of the city. Thus, the present study aimed at assessing the mortality burden attributable to long-term exposure to ambient PM_2.5_, PM_10_ and NO_2_ (above the updated AQG 2021 levels) during 2013–2017 in Ancona with a particular focus on potential differences in health impact according to the distance from the port.

## Materials and methods

### Study area

Ancona's city is extended over a 123 km^2^ area and had over 100,696 inhabitants in 2017. The city is subdivided into 722 census tracts by the National Institute of Statistics (ISTAT), i.e. the spatial unit considered in this study (approximatively 139 inhabitants each census tract).

Located in the middle of the Italian Adriatic coast, the port areas are 1.4 km2 articulated in passenger and ferry terminals, container and general cargo facilities. The port is crucial in the Adriatic-Ionian Macro-Region as the terminal of the international ferry routes to Greece, Croatia and Albania.

**Fig. 1:**
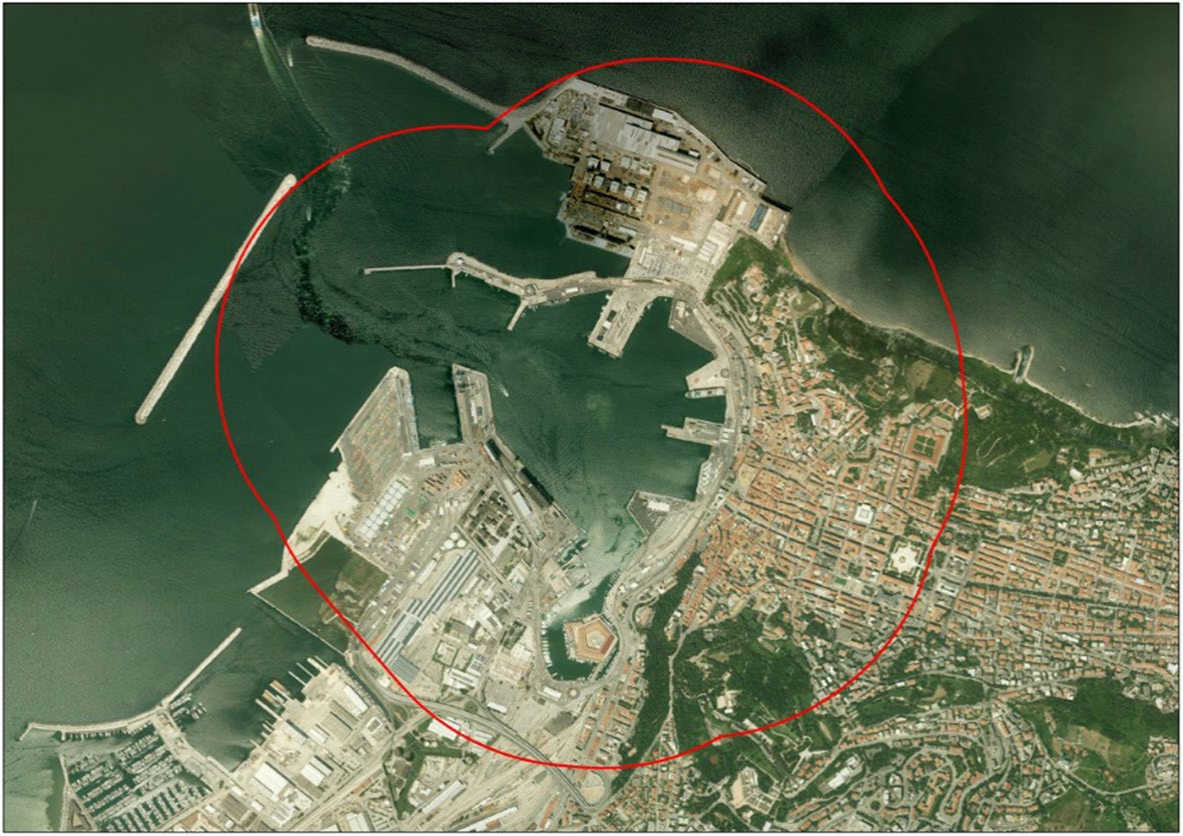
City map with 800 × 800 m buffer centered in the harbour area

As indicated, the harbour is located in the central part of the city and, to better characterize the harbour's health impact, we stratified the city into harbour areas/yes–no using an 800 m radius buffer from the centroid of the port, in order to select at least the 10% of total population, as shown in Fig. 1. The 800 m cut-off was decided a priori before the analysis was done and it was considered a good compromise to select an adequate number of people. The geographic spatial calculations were carried out with ArcGis software [11].

### Environmental data

**Fig. 2:**
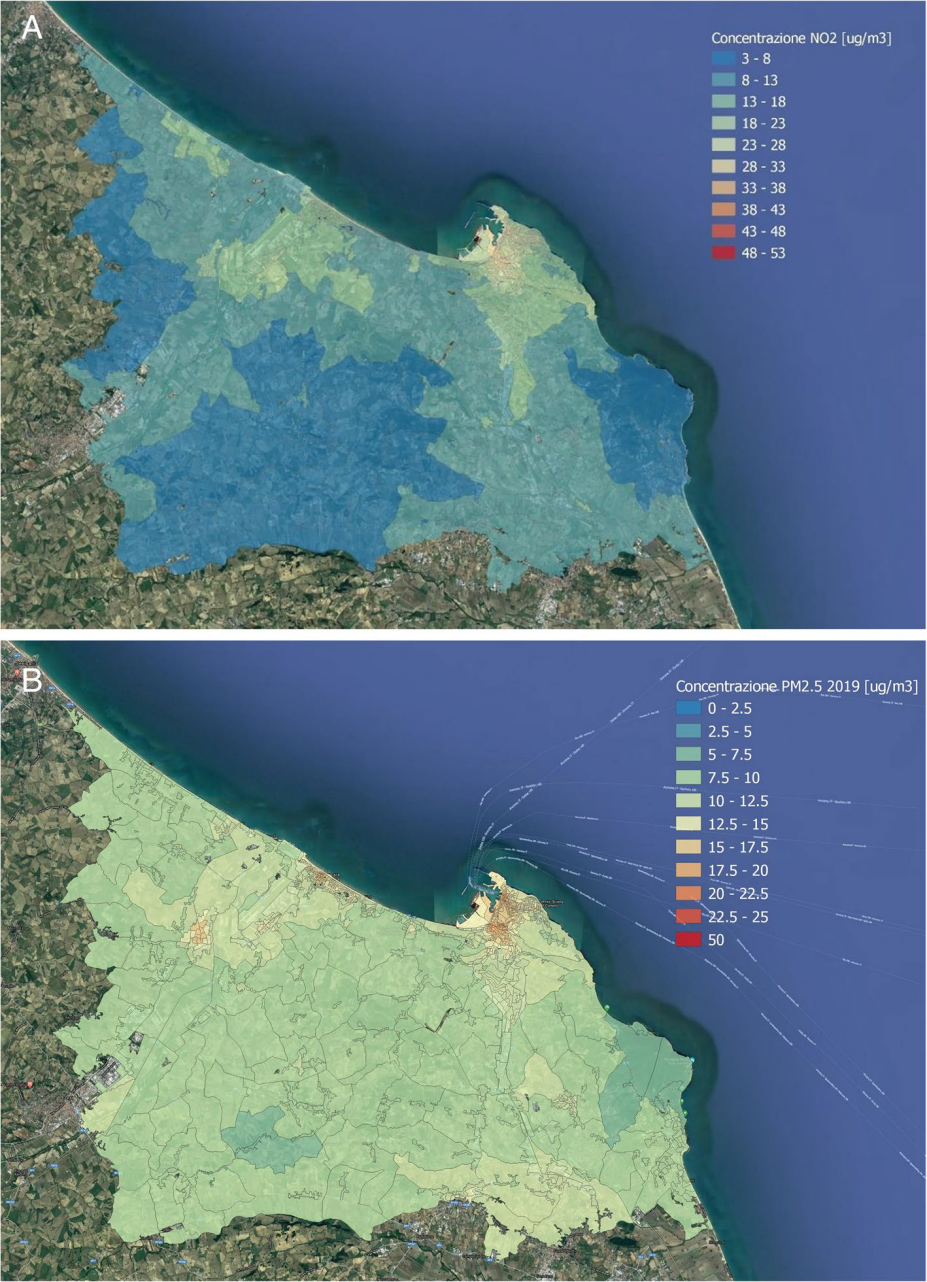
A-B. AverageNO_2_ (**A**) and PM_2._5 (**B**) annual concentrations in µg/m^3^ over the grid map of Ancona

Exposure to PM_10_, PM_2.5,_ and NO_2_ was derived by FARM (Flexible Air Quality Regional Model, www.farm-model.org) models on a 30 × 30 km spatial domain with a 0.5 × 0.5 km resolution provided by ARIANET, which are well described elsewhere [12, 13]. Briefly, FARM models are multi-grid Eulerian models, which account for the dispersion, transformation, and deposition of airborne pollutants in gas and aerosol phases by using meteorological and orographic information; subsequently, they are validated by observed data collected through fixed monitors. We assigned to each census tract specific exposure based on the 0.5 × 0.5 km grid map provided by ARIANET. For each spatial unit, we averaged the values of all cells overlapped and then weighted for the intersection area, as shown in Fig. 2 A-B. In our study, we used data based on 2019 as proxy of the total average exposure.

### Health data

We collected information for cause-specific mortality in each census tract of the city of Ancona. In detail, we obtained data for all-cause (International classification of diseases 10^th^ version ICD-10^th –^ A00 – R99), cardiovascular (I00-I99), respiratory (J00-J99), malignancies (C00-C99) and lung cancer (C34) mortality. For each event, we gathered information regarding age (in five classes: 0–4, 5–34, 35–64, 65–74, and 75 + years) and gender. Data were provided by the regional health information system of the Marche Region for the 2013–2017 period. Finally, data were aggregated for each census tract in the whole period. The socioeconomic indicator at the census tract level was derived from the Italian deprivation index based on the 2011 national census data about income, educational level and other socioeconomic covariates [14].

### Statistical analysis

Continuous variables were reported as mean values and standard deviations, while the categorical ones were reported as total numbers and percentages for each category. The relationship between the long-term effects of air pollution exposure and cause-specific mortality was evaluated using the population at the census tract level as the denominator of the events in Poisson regression models. Specifically, the exposure was represented by the average pollutants concentration for each census tract, while the outcome was the total count of events for each census tract. We applied three different models by the level of confounding adjustment: 1) crude association with no adjustment; 2) adjustment for age and sex; 3) model 2 + socioeconomic position indicator, which is an area index (census tract specific) synthesizing many social and economic variables. Moreover, we conducted an additional analysis to evaluate the association of living close to the city's harbour area with mortality.

Results are expressed as percent change of risk and relative 95% confidence intervals per 5 unit increase in air pollutants exposure.

Finally, we have computed the health impact assessment of long-term air pollution exposure on an annual number of premature cause-specific deaths. The method which we have used has been explained in detail elsewhere [15]. Briefly, we have applied the following formula:

$$\mathrm{ADi }=\mathrm{ TDi}*\mathrm{AFi}$$

where:

ADi is the number of attributable deaths; TDi is the number of total deaths; AFi is the attributable fraction, which is obtained by:

$$\mathrm{AF}=\left(\mathrm{RR}-1\right)=\left[\exp\{\mathrm\beta\ast\left(\mathrm{exposure}-\mathrm{AQG}\right\}-1\right]/\exp\left\{\mathrm\beta\ast\left(\mathrm{exposure}-\mathrm{AQG}\right)\right\}$$

The beta coefficient was extrapolated by evidence derived from systematic reviews and meta-analyses of several studies [3]. Specifically, we considered the following relative risks per per 10 µg/m^3^ PM_2.5_: 1.08 (1.06, 1.09), 1.11 (1.09, 1.14), 1.10 (1.03, 1.18) and1.12 (1.07, 1.16) for non-accidental, cardiovascular, respiratory and lung cancer mortality, respectively. In this way, we obtained the number of premature deaths that would have been prevented if the WHO AQG had been adopted in the study setting.

Analyses were conducted using the statistical software R [16].

## Results

Table 1 reports the population characteristics in the city of Ancona during the study period 2013–2017, subdivided according to the area of the city. Almost 10% of the subjects live in the city's harbour area, and 75% are less than 65 years old. Population demographics and socioeconomic status did not differ between the harbour and non-harbour areas. Higher PM_2.5_ and NO_2_ concentrations were estimated in the area close to the port than in the rest of the city: 21 vs 18.1 and 25.5 vs 18.4 µg/m^3^, respectively. In the lustrum, we observed 5,584 deaths, among which 2,051 were due to cardiovascular causes and 1,594 due to malignancies. No substantial differences emerged in the distribution of individual characteristics or deaths between the two areas.

**Table 1:** Population characteristics and the number of cause-specific deaths by area of the city during the study period 2013–2017

	**Harbour area**				**Total**
	No		Yes		
	N (mean)	% (SD)	N (mean)	% (SD)	
**Total population**	90,085	89.8	10,258	10.2	100,343
**Exposure**					
PM_2.5_	18.1	4.7	21	1.5	
NO_2_	18.4	6.3	25.5	2.1	
**Age class**					
0–4	3,838	4.3	491	4.8	4,329
5–34	25,406	28.2	3,088	30.1	28,494
35–64	38,217	42.4	4,466	43.5	42,683
65–74	10,677	11.9	928	9	11,605
75 +	11,947	13.3	1,285	12.5	13,232
**Sex**					
Females	47,377	52.6	5,439	53	52,816
Males	42,708	47.4	4,819	47	47,527
**Socioeconomic position**					
Very low	9,836	10.9	1,140	11.1	10,976
Low	10,770	12	1,501	14.6	12,271
Medium	13,048	14.5	1,892	18.4	14,940
High	19,437	21.6	1,752	17.1	21,189
Very high	31,470	34.9	3,823	37.3	35,293
Missing	5,524	6.1	150	1.5	5,674
**Mortality**					
Natural	5,003	5.6	581	5.7	5,584
Cardiovascular	1,817	2	234	2.3	2,051
Respiratory	347	0.4	48	0.5	395
Cancer	1,444	1.6	150	1.5	1,594
Lung cancer	257	0.3	21	0.2	278

*Footnotes*: *SD* standard deviation NO_2_: nitrogen dioxide, *PM* particulate matter

In Table 2, we describe the results of the association between long-term exposure to air pollution and cause-specific mortality in the city of Ancona during 2013–2017. We observed a positive association between each pollutant and all considered outcomes, except for respiratory and cancer mortality. In the model 3 analyses, the estimates were up to 7.6% (95%CI: 0.1, 15.6%) for NO_2_ and cardiovascular mortality and 15.3% (95%CI: -1.1, 37.2%) for the association between PM_2.5_ and lung cancer. We observed lower positive estimates for natural mortality but with large confidence intervals.

**Table 2:** Association between long-term exposure to PM_10_, PM_2.5_ and NO_2_ and cause-specific mortality in the city of Ancona during 2013–2017 period using different levels of adjustment

**Mortality**		**Model 1**^**a**^			**Model 2**^**a**^			**Model 3**^**a**^		
	**Pollutant**	**%change**	**95%CI**		**%change**	**95%CI**		**%change**	**95%CI**	
**Natural (5,584)**	PM_10_	13	4.6	22.6	2.3	-0.8	5.6	1.9	-1.2	5.2
	PM_2.5_	17.5	5.9	31.5	3.1	-1.1	7.5	2.5	-1.6	6.9
	NO_2_	15.6	4.5	28	3.9	-0.5	8.6	3.1	-1.3	7.7
**Cardiovascular (2,051)**	PM_10_	18.8	7	32.9	4.9	-0.3	10.7	4.8	-0.5	10.6
	PM_2.5_	25.9	9.2	47.2	6.8	-0.3	14.9	6.5	-0.6	14.6
	NO_2_	23.2	7.8	40.8	8.1	0.6	16.2	**7.6**	**0.1**	**15.6**
**Respiratory (395)**	PM_10_	10	-7.8	35.3	-0.5	-9.2	10.1	-1	-9.7	9.5
	PM_2.5_	13.6	-10.1	51.5	-0.3	-11.6	14.2	-1.3	-12.4	13.2
	NO_2_	14.5	-10.9	47.2	2.8	-10.4	18	0.9	-12.2	16
**Cancer (1,594)**	PM_10_	6.2	-2.4	16.4	0.2	-4.9	5.8	-0.3	-5.3	5.3
	PM_2.5_	8.1	-3.3	22.5	0.2	-6.3	7.8	-0.4	-6.9	7
	NO_2_	6.7	-5.2	20.1	0.2	-7	8.1	-0.4	-7.6	7.4
**Lung cancer (278)**	PM_10_	17.1	-2.5	44.7	13.8	1.3	29.4	11.9	-0.2	26.9
	PM_2.5_	22.8	-3.9	65	18.3	1.1	41.2	15.3	-1.1	37.2
	NO_2_	16.5	-9	49.1	13.8	-2.7	33.1	11.1	-5	29.9

The results are expressed as percent change of risk and relative 95% confidence interval (95%CI) per 5 mg/m^3^ increases in the pollutants

*Footnotes*: *NO2* nitrogen dioxide, *PM* particulate matter

^a^Model 1: crude Association with no adjustment; Model 2: adjustment for age and sex; Model 3: model 2 + socioeconomic position indicator

In Table 3, we show the results related to subjects who lived in the city's central area close to the harbour, in comparison to subjects residing elsewhere. We observed higher risks of mortality for natural, cardiovascular and respiratory causes for those living in the central part of Ancona. In detail, there was an excess risk of up to 13.5% (95% CI, 4.2–23.7) for natural mortality, up to 24.1% for cardiovascular mortality (95% CI, 8.3–42.3), and up to 37.9% (95% CI, 7.5–76.95) for respiratory mortality.

**Table 3:** Association (%change) between residence in central area of the city close to the harbour and cause-specific mortality (2013–2017)

Mortality	Model1^a^			Model2^a^			Model3^a^		
	**%change**	**95%CI**		**%change**	**95%CI**		**%change**	**95%CI**	
Natural (5,584)	7.2	-12.6	31.6	13.3	3.9	23.5	**13.5**	**4.2**	**23.7**
Cardiovascular (2,051)	18.9	-8.2	54.0	24.0	8.2	42.2	**24.1**	**8.3**	**42.3**
Respiratory (395)	27.7	-20.2	104.4	37.2	6.8	76.1	**37.9**	**7.5**	**76.9**
Cancer (1,594)	-4.1	-25.2	23.1	3.2	-11.6	20.5	3.3	-11.6	20.7
Lung cancer (278)	-24.5	-58.2	36.3	-16.6	-42.2	20.4	-16.0	-41.7	21.1

^a^Model 1: crude Association with no adjustment; Model 2: adjustment for age and sex; Model 3: model 2 + socioeconomic position indicator

**Fig. 3:**
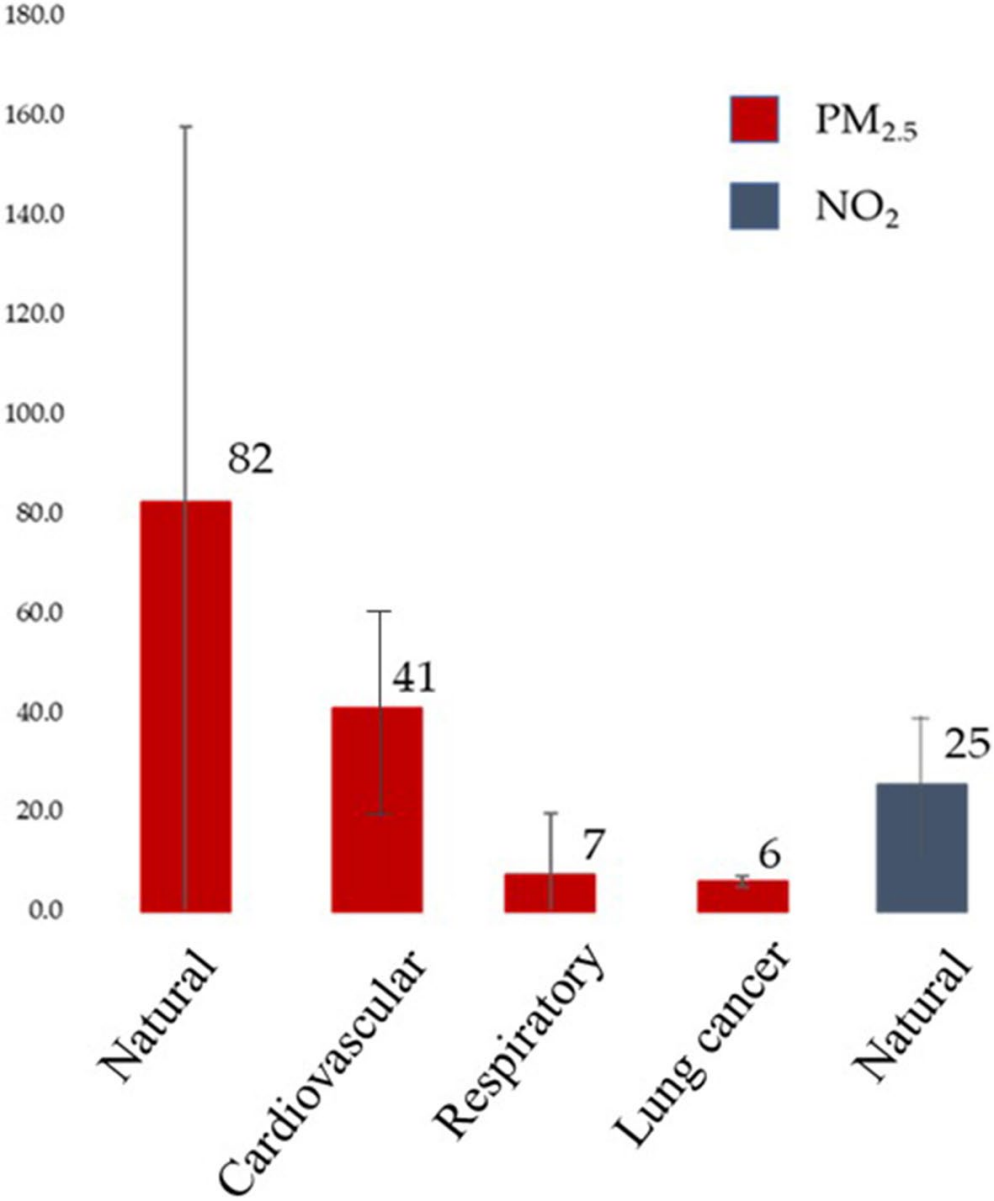
Annual preventable premature deaths due to long-term exposure to PM_2.5_ and NO_2_ in the city of Ancona

Finally, Fig. 3 reports the number of annual premature deaths which would be preventable if the yearly exposure to PM_2.5_ and NO_2_ were compliant with the AQG: 82 and 25 total annual deaths due to the excess of PM_2.5_ and NO_2_ exposure, respectively.

## Discussion

Our study was performed through a record linkage between healthcare databases and environmental data. The use of an accurate dispersion model provides evidence of positive associations between long-term exposure to major air pollutants and cause-specific mortality in the city of Ancona during the 2013–2017 period. The strongest association was observed for NO_2_ and cardiovascular-related mortality (7.6% increase per 5 µg/m^3^). All the associations are in keeping with the findings from the systematic reviews conducted to derive the WHO AQGs. A further relevant and innovative finding of our study is the significantly increased natural and cause-specific mortality risks observed in subjects living in the city's central area close to the harbour, where PM_2.5_ and NO_2_ annual mean exposure levels were largely above the latest AQG thresholds. Although studies on shipping emissions impact on human health have been increasing in recent years, using different developed methods [10], this is the first investigation specifically addressing the health burden in the urban area closest to the port-related emissions, showing an excess mortality risk. Overall, although our estimates have wide confidence interval, yet 82 premature deaths from PM_2.5_ exposure and 25 premature deaths from NO_2_ exposure per year would have been avoided if WHO AQGs had been respected.

The impact of exposure to major air pollutants on natural and selected cause-specific mortality in Ancona has already been investigated in a multi-center study on short-term exposures involving 25 Italian cities (EpiAir2) but the different methods and sources employed for analyses make it difficult to compare the results [17].

Several epidemiological studies have correlated increased air pollution to cardiovascular events, such as arrhythmias, atherosclerosis progression, myocardial infarction, and heart failure exacerbation [3, 18, 19]. Almost two-thirds of deaths attributed to pollution are due to cardiovascular diseases, outweighing mortality due to respiratory conditions, and 33% of global cardiovascular deaths are currently attributable to air pollution [1, 20]. Plausible pathogenic pathways include endothelial dysfunction and vasoconstriction, increased blood pressure, prothrombotic and coagulant changes, systemic inflammatory and oxidative stress responses, and autonomic imbalance [21–23].

It is also well established that increased exposures to various air pollutants contribute to exacerbations in patients with chronic respiratory diseases, such as asthma and chronic obstructive respiratory disease [4, 24]. The respiratory tract is the primary portal of entry of PM; thus, airway inflammation, remodeling, and responsiveness are likely to be promoted and amplified by both acute and chronic inhalation of these irritants [4].

The association between PM_2.5_ and lung cancer has also been investigated in lifelong non-smokers to remove the confounding role of active smoke. Lung cancer incidence in this subgroup has been rapidly increasing over the recent decades, accounting for approximately 25% of cases worldwide, and PM_2.5_ is currently deemed to play a critical role in this context [25]. Data from 17 cohort studies in nine European countries (ESCAPE) showed that a 5 μg/m^3^ increment in annual exposure to PM_2.5_ was associated with an increased hazard ratio of lung cancer incidence with a relative risk of 1.18 [26]. The relative risk per 10 μg/m^3^ increase in PM_2.5_ for development of lung cancer was 1.19 (95% CI: 1.09, 1.30) in a large study in the USA [27]. Furthermore, an increase in PM_2.5_ concentration was positively correlated with an increase in lung cancer mortality. Indeed, outdoor PM_2.5_ pollution was defined as a Group 1 human carcinogen by the International Agency for Research on Cancer (IARC) in 2013 [28, 29]. Potential pathogenic carcinogenesis mechanisms include inflammatory injury, reactive oxygen species production, and oxidative damage to DNA. Our study detected an increased risk of lung cancer, although with a large confidence interval.

With reference to source-specific emission effects, while numerous studies looked at road transport-sourced air pollution, relatively less data are available on shipping and port-related health impact. However, over the last decade, the ability to assess the health effects and the chemical and physical characteristics of ship pollutant exposure were identified as key knowledge gaps and, thus, a growing attention has been given to this challenging context.

A major strength of the present paper, as mentioned above, is the assessment of air pollutant concentration in a city characterized by the presence of a large harbor located in the central urban area, analyzing natural and cause-specific mortality risk overall and separately for subjects living in zones near to this source. However, some limitations need to be acknowledged. Data on air pollutant concentrations at spatial level were the results of a model, although validated through a local monitoring unit. Moreover, due to the lack of measurement stations in the harbor area, it was not clear what proportion of air pollution was actually shipping-sourced and to what extent emissions from non-ship activities, such as cargo-loading machinery, heavy-duty vehicles or trains have influenced the overall burden. Furthermore, we used the average exposure of the year 2019 assuming that no spatial differences emerged during the study period. Another limitation could be related to the arbitrary a priori choice of 800 m cut-off for buffer. However, a sensitivity analysis considering different buffers related to the central area was performed (600m^2^ and 1000m^2^ buffers), showing consistent results among the three buffers (data not shown), but confirming the 800 m^2^ one as the best compromise between an adequate size of population exposed and weight of exposure.

Considering that Ancona is a relatively small city, the limited number of cause-specific events observed may have impacted the power of the study and the generalizability of the results. In fact, the observed associations are characterized by high uncertainty, with wide confidence intervals; in addition, they are often of borderline significance. A further limitation is related to the data sources: administrative databases not primarily designed as tools for epidemiological research. As a result, information on important individual risk factors, such as smoking habits, physical activity, and diet, was unavailable. Therefore, some degrees of residual confounding might be possible in the evaluation of the associations, especially when comparing the harbour area with the rest of the city. It should be considered, however, that we adjusted our models for socioeconomic status, which is a proxy of some lifestyle behaviours including those previously described. In this way, we were able to take into account, at least partially, the role of some individual risk factors [30, 31]. We did not consider other variables, like access and quality of health care that, in theory, are guaranteed to all citizens in Italy under the National Health Service. In a small town like Ancona, we have no reasons to think that healthcare is different in different parts of the city.

## Conclusion

Our analysis showed a significant long-term health effect of PM and NO_2_, indicating a not negligible higher mortality burden in areas close to a substantial source of air pollutants like the port. Considering that a large majority of the world's population currently lives in areas where air pollution exceeds AQG levels, especially those with intense industrial/portal activity, it is essential to locally estimate the concentration of air pollutants and to urgently adopt targeted policy action to reduce the mortality burden attributable to the air quality. Estimating the source-specific health burdens is key to achieve a deeper understanding of the role of different emission sources, as well as to support the design of effective and targeted mitigation strategies.

## Data Availability

The datasets used during the current study was available from the corresponding authors.
